# Geometric Model and Calibration Method for a Solid-State LiDAR

**DOI:** 10.3390/s20102898

**Published:** 2020-05-20

**Authors:** Pablo García-Gómez, Santiago Royo, Noel Rodrigo, Josep R. Casas

**Affiliations:** 1 Centre for Sensors, Instrumentation and Systems Development, Universitat Politècnica de Catalunya (CD6-UPC), Rambla de Sant Nebridi 10, 08222 Terrassa, Spain; santiago.royo@upc.edu (S.R.); noel.rodrigo@upc.edu (N.R.); 2 Beamagine S.L., Carrer de Bellesguard 16, 08755 Castellbisbal, Spain; 3 Image Processing Group, TSC Department, Universitat Politècnica de Catalunya (UPC), Carrer de Jordi Girona 1-3, 08034 Barcelona, Spain; josep.ramon.casas@upc.edu

**Keywords:** solid-state LiDAR, LiDAR calibration, distortion correction, FOV mapping

## Abstract

This paper presents a novel calibration method for solid-state LiDAR devices based on a geometrical description of their scanning system, which has variable angular resolution. Determining this distortion across the entire Field-of-View of the system yields accurate and precise measurements which enable it to be combined with other sensors. On the one hand, the geometrical model is formulated using the well-known Snell’s law and the intrinsic optical assembly of the system, whereas on the other hand the proposed method describes the scanned scenario with an intuitive camera-like approach relating pixel locations with scanning directions. Simulations and experimental results show that the model fits with real devices and the calibration procedure accurately maps their variant resolution so undistorted representations of the observed scenario can be provided. Thus, the calibration method proposed during this work is applicable and valid for existing scanning systems improving their precision and accuracy in an order of magnitude.

## 1. Introduction

Nowadays, Light Detection and Ranging (LiDAR) devices are aimed to be used in a wide variety of applications among which autonomous vehicles and computer vision for robotics are outstanding. Their first studies and uses were related to atmospheric observations [1,2,3] and airborne mapping [4,5] decades ago. Progressively, the 3D sensing capability of LiDARs pushed them towards more user-oriented devices at the same moment in which audiovisual and computer vision applications such as object detection, RGB + depth fusion and augmented reality emerged [6,7].

Furthermore, the current disruption of autonomous driving and robotics has forced LiDAR technology to move a step forward in order to meet their demanding specifications: large range and high spatial resolution whilst real-time performance and background solar tolerance. Initially, mechanical rotating LiDARs [8] showed up as the solution for this technical challenge as they were based on sending light pulses instead of using amplitude-modulated illumination, prone to problems with background illumination outdoors. Mechanical LiDARs do imaging through spinning a macroscopic element, either the whole sensor embodiment or an optical element such as a prism or a galvanometer mirror [9]. Nonetheless, moving parts generally mean large enclosures and poor mechanical tolerance to vibration, shock and impact. As a consequence, and although they have been widely used in research and some industrial applications [10,11,12], solid-state LiDARs which precisely avoid large mechanical parts have arisen great interest because they also provide scalability, reliability and embeddedness.

Currently, several scanning strategies avoiding moving parts are emerging. Due to their constant evolution, there is a vast variety of literature, either scientific publications or patents, concerning how the scanning method is performed. However, there is no detailed description on how 3D data is actually calculated.

Since new computer vision techniques require accurate and precise 3D representations of the surroundings of the object for critical applications, there is a real need to provide models and calibration algorithms able to correct or compensate any possible distortion in LiDAR scanning. Adding the fact that research is yet mainly based on mechanical techniques, there is a lack of calibration procedures for non-mechanical, solid-state devices, in contrast to rotating ones [13,14,15,16,17,18,19]. Up to date, significant research about LiDAR imaging assumes the LiDAR device provides (x,y,z) data precise enough from the direct measurement [20,21,22,23,24,25,26].

Thus, the main aim of this work is to present a general-purpose, suitable and understandable scanning model along with a feasible calibration procedure for non-mechanical, solid-state LiDAR devices. In order to do so, the geometry of the system and the sources of distortion will be discussed during the second section of the paper. Afterwards, the third section will describe the model and the calibration algorithm proposed as well as the LiDAR devices used for this work. Then, the fourth section will show the results of the implementation of the suggested method for both devices whereas the last section will gather the presented results and previous ones for comparison and for reaching conclusions about this work. Two appendixes have been added with the detailed derivation of some of the equations discussed.

## 2. Problem and Model Formulation

Imaging LiDARs are based on measuring the Time-of-Flight (TOF) value for a number of points within their Field-of-View (FOV). Then, the whole set of points, usually known as point cloud, becomes a 3D representation of the scenario being observed. Without prejudice to the generality and keeping the broad variety of techniques for either obtaining the TOF measurement and performing the scanning, the nth measured point $p_{n}$ in the LiDAR reference system {L} can be expressed as follows:

(1) $${}^{\left\{L\right\}}p_{n}\equiv {\left[\begin{array}{ccc} {}^{\left\{L\right\}}x_{n}\;, & {}^{\left\{L\right\}}y_{n}\;, & {}^{\left\{L\right\}}z_{n} \end{array}\right]}^{T}=\frac{c}{2}t{}_{TOF,n}\cdot {}^{\left\{L\right\}}{\hat{s}}_{n},$$

where *c* is the speed of light in air, $t_{TOF,n}$ is its TOF measure and ${\hat{s}}_{n}$ is the unitary vector representing the scanning direction of the LiDAR described in its reference system {L}. From this equation, it can be appreciated that both the TOF measurement $t_{TOF,n}$ and the scanning direction ${\hat{s}}_{n}$ are crucial for obtaining accurate and precise point clouds.

The goal of this paper is to provide a method for characterizing the whole FOV of a solid-state LiDAR system in order to obtain more accurate and precise point clouds through mapping its angular resolution in detail. As a consequence, this section in particular is going to focus on the scanning direction of an imaging LiDAR system.

Firstly, the imaging distortion causes will be presented by focusing on a particular solid-state LiDAR system, which uses a two-axis micro-electrical-mechanical system (MEMS) mirror [27] as a reflective surface in order to aim the light beam in both horizontal and vertical directions by steering on its two axis. Therefore, the mechanics and the dynamics of the scanning system are analyzed on the basis of vectorial Snell’s law which, eventually, leads us to a non-linear expression relating the two tilting angles of the MEMS with the scanning direction ${\hat{s}}_{n}$. Comparable approaches for obtaining the non-linear relation between the scanning direction and its scanning principle may be taken for other scanning methods.

After presenting the distortion causes, a general description of the LiDAR imaging system is going to be presented. It is based on a spherical description of the scanning direction and it is suitable for a broad variety of scanning techniques. This description will be used on the following sections to reach the goal of this paper. Let us start addressing the problem of FOV distortion then.

### 2.1. The Problem

Ideally, the FOV of the system corresponding to the set of all ${\hat{s}}_{n}$ should have constant spacing, meaning constant spatial resolution. Nonetheless, such spacing is distorted as a result of varying resolution from either optical and/or mechanical mismatch, no matter how the scanning is performed. Thus, a characterization of these undesired artifacts must be done. Generally, it is done using ray tracing based in the well-known Snell’s law in combination with some geometrical model of the scanner [14,16,18] as previously introduced.

Snell’s law describes how light is reflected and transmitted at the interface between two media of different refractive indices. Thus, it is used in optics to compute those angles and estimate light paths [28]. Commonly, it is expressed as a scalar function so a 2D representation is obtained. However, there is an important assumption behind it—the incident and the reflected and/or transmitted rays lay in the same plane of the surface normal. Consequently, one of them is a linear combination of the other two vectors. Let us describe the reflected ray $\hat{r}$ as a linear combination of the incident ray $\hat{i}$ and the surface normal $\hat{n}$, all of them being unitary vectors.

(2) $$\hat{r}=\mu \hat{i}+\eta \hat{n}.$$

**Figure 1:**
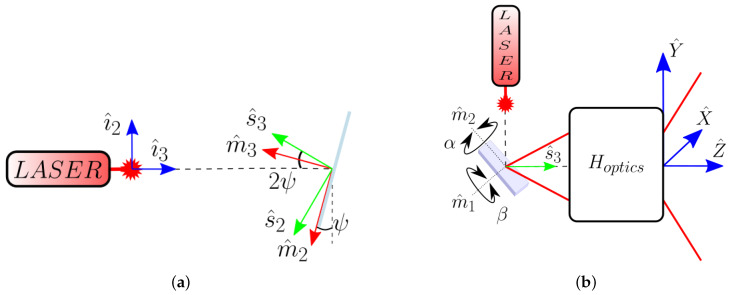
Scheme of (**a**) the Light Detection and Ranging (LiDAR) scanning system geometry depicting (**blue**) the laser source, (**red**) the MEMS mirror and (**green**) the scanning reference systems with ${\hat{i}}_{1}={\hat{m}}_{1}={\hat{s}}_{1}$ pointing inside the paper plane; and (**b**) the global geometry of the LiDAR scanning setup in the general case, with the tilt angles of the MEMS magnified by additional optics.

Vectorial Snell’s law is obtained from the above equation imposing the scalar law and using vectorial algebra in conjunction with the previously commented assumption. (A detailed derivation of Equation (3) may be found in Appendix A.1.)

(3) $$\hat{r}=\mu \hat{i}-\left(\mu \left(\hat{n}\cdot \hat{i}\right)-\sqrt{1-\mu^{2}\left(1-{\left(\hat{n}\cdot \hat{i}\right)}^{2}\right)}\right)\hat{n},$$

where η in Equation (2) is the expression between brackets and $\mu =n_{i}/n_{r}$ is the ratio between the refractive indices of the incident and reflected media. Notice that in general μ=1 since both incident and reflected media are the same and usually air. Despite this simplification, the expression is non-linear due to the intrinsic $\left(\hat{n}\cdot \hat{i}\right)$ dependence of η. Hence, the non-linearity on the surface normal is the origin of the optical distortion of the FOV as the beam steering is performed by tilting the reflective surface. In addition, and according to Figure 1b which is explained below, there is commonly an optical assembly placed, called a field expander, in order to obtain larger FOVs. As a consequence, each lens surface interacts with the scanning direction following Equation (3), which introduces additional distortion as well.

Let us now use geometric algebra to express how the tilt angles of the MEMS rotate the surface normal $\hat{n}$, and consequently, the reflected ray $\hat{r}$ using Equation (3) which is the scanning direction. The above Figure 1 shows the set of reference systems and angles used in the geometrical description of the LiDAR—the reference system of the laser source {I}, the one for the MEMS or any reflective surface at its resting or central position {M}, and the one for the scanning optics {S}.

Appendix A.2 includes a detailed derivation of the relationships between them resulting in Equation (A13). It leads to the following expression for the LiDAR scanning direction $\hat{s}$ in the laser source reference system {I} as a function of the tilt angles of the mirror α and β, for the horizontal and vertical directions respectively, which may be found to follow Equation (3).

(4) $$\begin{array}{cc} \; & {}^{\left\{I\right\}}\hat{s}=\;{}^{\left\{I\right\}}\hat{i}-\left(\gamma \left(\alpha ,\beta ,\psi \right)-\sqrt{\gamma {\left(\alpha ,\beta ,\psi \right)}^{2}}\right)\;{}^{\left\{I\right\}}\hat{n}\\ \; & \mathrm{with}\;{}^{\left\{I\right\}}\hat{n}=\left[\begin{array}{c} \sin\left(\alpha \right)\cos\left(\beta \right)\\ \cos\left(\psi \right)\sin\left(\beta \right)+\sin\left(\psi \right)\cos\left(\alpha \right)\cos\left(\beta \right)\\ \sin\left(\psi \right)\sin\left(\beta \right)-\cos\left(\psi \right)\cos\left(\alpha \right)\cos\left(\beta \right) \end{array}\right]\\ \; & \mathrm{and}\;\gamma \left(\alpha ,\beta ,\psi \right)={}^{\left\{I\right\}}{\hat{n}}_{3}=\sin\left(\psi \right)\sin\left(\beta \right)-\cos\left(\psi \right)\cos\left(\alpha \right)\cos\left(\beta \right), \end{array}$$

where, as expressed in the last row, γ is just the third component of the normal vector $\hat{n}$ expressed in the laser source reference system {I}. It depends on the scanning tilt angles α and β as well as the angle between the incident direction of the laser beam and the mirror surface ψ. It can be seen that Equation (4) makes even more explicit the non-linear relationship between the scanning direction $\hat{s}$ and the tilt angles α and β.

At this point, we can consider the effect of the scanning dynamics of the surface normal $\hat{n}$ due to the continuous tilt of the surface. Imaging at high frame rates is an essential requirement for the considered applications. Here it remains one of the key advantages of using MEMS mirrors. Because of being micro devices with large surface area to volume ratios, they offer less resistance to motion and, more importantly, electromagnetic and fluid forces are more relevant than other physical forces such as torques. The overall effect is that they can be rapidly driven with fast electrical signals providing lower inertia values so they are able to keep clearer and well-defined dynamics at larger frequencies [27].

In order to reach higher frame rates, one of the tilt angles is usually driven at a fast oscillation frequency near its resonance. As a consequence, its motion is no longer linear, not even harmonic, and it becomes asymmetric, resulting in an additional distortion of the angular resolution as further discussed in Section 3.1.

In summary, the distortion of the FOV is an unavoidable consequence of how the scanning is performed due to two key aspects. The first one resides in the optical nature of LiDAR devices, here demonstrated using Snell’s law. Additionally, the dynamics of the beam steering technique also introduces an important variation on the spatial resolution of the system. Thus, the following sections will provide the model and the method for characterizing the whole FOV of a solid-state LiDAR system through mapping its angular resolution in detail.

### 2.2. The Model

In order to do so, let us start by defining the LiDAR reference system. Up to now, three different reference systems have been defined within the device as a result of applying rotation matrices. Taking into account that the scanning reference system {S} results from a rotation of the laser source system {I}, which was defined matching the Cartesian coordinates, it can be stated that {S} is an orthonormal basis of $R^{3}$. Then, any point within this Euclidean space can be expressed as a linear combination of the three basis of {S}. As a consequence, the LiDAR reference system $\left\{L\right\}=\left\{{\hat{L}}_{1},{\hat{L}}_{2},{\hat{L}}_{3}\right\}$ is defined as the scanning reference system {S} and, for convenience, centered at the origin of the space.

**Figure 2:**
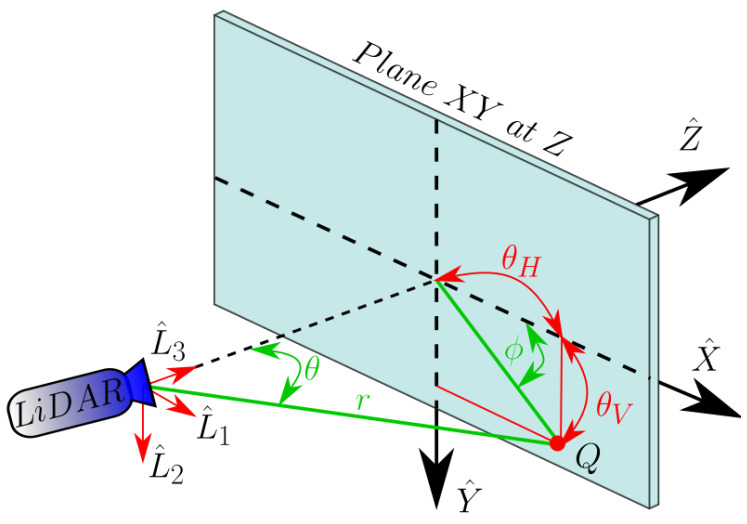
Scheme of the LiDAR Field-of-View (FOV) and an observed point **Q** relating (**red**) the LiDAR reference system and its scanning angles (**green**) in spherical coordinates.

Given a target point Q with known (X,Y,Z) coordinates, its expression in LiDAR coordinates is then ${}^{\left\{L\right\}}Q=X{\hat{L}}_{1}+Y{\hat{L}}_{2}+Z{\hat{L}}_{3}$ as Figure 2 represents. Since TOF resolves the euclidean distance to this point, the most useful coordinate system is the spherical one. Thus, the point could be expressed as the previous Equation (1), ${}^{\left\{L\right\}}Q=\left(\frac{c}{2}t{}_{TOF}\right){\left[\begin{array}{ccc} \sin\left(\theta \right)\cos\left(\phi \right), & \sin\left(\theta \right)\sin\left(\phi \right), & \cos\left(\theta \right) \end{array}\right]}^{T}$.

Let us now relate the polar angle θ and the azimuthal angle ϕ with the FOV of the system. Using trigonometry, both horizontal and vertical viewing angles can be related to (X,Y,Z).

(5) $$\theta_{H}\equiv \theta_{X}=\arctan\left(\frac{X}{Z}\right)\;\mathrm{and}\;\theta_{V}\equiv \theta_{Y}=\arctan\left(\frac{Y}{Z}\right).$$

From the previous definition of {L}, θ is the angle between the vector going from the center of the reference system to Q and the LiDAR’s optical axis ${\hat{L}}_{3}=k$. Thus:

(6) $$\theta =\arctan\left(\frac{\sqrt{X^{2}+Y^{2}}}{Z}\right)=\arctan\left(\sqrt{{\left(\frac{X}{Z}\right)}^{2}+{\left(\frac{Y}{Z}\right)}^{2}}\right)=\arctan\left(\sqrt{\tan{\left(\theta_{H}\right)}^{2}+\tan{\left(\theta_{V}\right)}^{2}}\right).$$

Both viewing angles range from $\left[-{\mathrm{FOV}}_{i}/2,{\mathrm{FOV}}_{i}/2\right]$, being ${\mathrm{FOV}}_{i}$ the respective horizontal or vertical FOV of the device, ${\mathrm{FOV}}_{H}$ and ${\mathrm{FOV}}_{V}$. It can be demonstrated that for |x|<π/2 both $\arctan\left(x\right)∼x-x^{3}/3+x^{5}/5+O\left(x^{7}\right)$ and $\tan\left(x\right)∼x+x^{3}/3+2x^{5}/15+O\left(x^{7}\right)$ functions are below their real value. Hence, taking the first order for each expansion, the polar angle range is $\theta \in \left[0,\sqrt{{\left({\mathrm{FOV}}_{H}/2\right)}^{2}+{\left({\mathrm{FOV}}_{V}/2\right)}^{2}}\right)$. Consequently, the azimuthal angle is simply the arc-tangent between the horizontal and the vertical scanning angles, ranging from ϕ∈[0,2π). To sum up, the final expression for any point $p\in L\subseteq R^{3}$, being L the subset of points contained in the LiDAR’s full FOV, related to the viewing angles $\theta_{H}$ and $\theta_{V}$ is as follows:

(7) $$\begin{array}{cc} \; & {}^{\left\{L\right\}}p=\left(\frac{c}{2}t{}_{TOF}\right){\left[\begin{array}{ccc} \sin\left(\theta \right)\cos\left(\phi \right), & \sin\left(\theta \right)\sin\left(\phi \right), & \cos\left(\theta \right) \end{array}\right]}^{T}\\ \; & \mathrm{with}\;t{}_{TOF}\geq 0\\ \; & \mathrm{and}\;\theta =\arctan\left(\sqrt{\tan{\left(\theta_{H}\right)}^{2}+\tan{\left(\theta_{V}\right)}^{2}})\right)\in \left[0,\sqrt{{\left({\mathrm{FOV}}_{H}/2\right)}^{2}+{\left({\mathrm{FOV}}_{V}/2\right)}^{2}}\right)\\ \; & \mathrm{and}\;\phi =\arctan\left(\frac{\theta_{V}}{\theta_{H}}\right)\in \left[0,2\pi \right). \end{array}$$

## 3. Materials and Methods

In this section, we present the method with the scope of mapping both viewing angles $\theta_{H}$ and $\theta_{V}$ in order to characterize the varying angular resolution discussed in the previous sections and to provide reliable point clouds using Equation (7), as well as the tested devices and the calibration target.

### 3.1. Calibration Method

**Figure 3:**
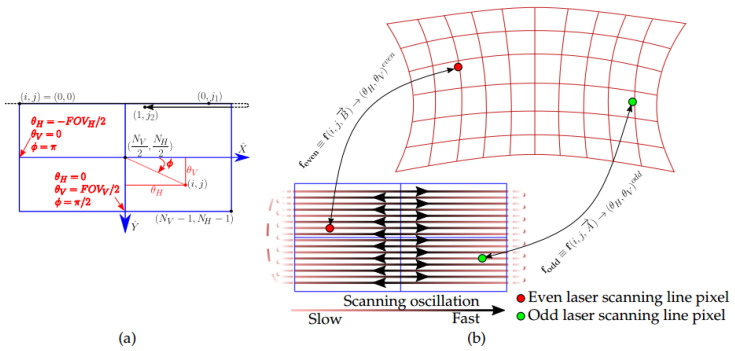
(**a**) Scheme of the LiDAR’s acquisition frame showing the relation between (**black**) the pixel position (*i*,*j*) from the MEMS dynamics and (**red**) the scanning angles of the FOV. (**b**) Scheme of the distortion mapping functions for odd and even lines, $f_{\mathrm{odd}}$ and $f_{\mathrm{even}}$, relating the pixel position within the acquisition frame with the final scanning angles.

Since the intensity of the back-reflected pulse can also be measured, let us consider a frame of the scanning optics as an image from now on. With this approach, each scanned direction becomes a pixel of it as depicted in Figure 3. Ideally, the angular resolution from one pixel to the next Δθ would be constant across the whole FOV so the viewing angles $\theta_{H}$ and $\theta_{V}$ for a pixel at row and column (*i*,*j*) would be simply linear as follows:

(8) $$\theta_{H}\left(j\right)=\left(j-\frac{N_{H}}{2}\right)\frac{FOV_{H}}{N_{H}}=\left(j-\frac{N_{H}}{2}\right)\Delta \theta_{H}\;\mathrm{and}\;\theta_{V}\left(i\right)=\left(i-\frac{N_{V}}{2}\right)\Delta \theta_{V},$$

where $N_{H}$ and $N_{V}$ represent the number of columns and rows, horizontal and vertical measurements, respectively.

Nonetheless, it has already been demonstrated that the FOV is distorted so non-linear terms must be wisely added. Traditionally, optical distortion has been modelled as a combination of radial and tangential distortion [29,30,31]. In the case of the LiDAR scanning, however, not only these cross-correlated optical terms play a role but also the dynamics of the MEMS scanning, as previously discussed. Generally, laser pulses are not sent when the oscillation is at its edges so the majority of its linear regime is seized, as it can be appreciated in Figure 3.

Moreover, radial symmetry is lost, so a proper distortion model is needed. For convenience, the fastest scanning direction, the horizontal, is split up by its two directions of motion in order to separate the strongest asymmetric behaviour of the MEMS. Thus, odd and even lines of the image are treated independently as two separate images yielding two independent distortion mapping functions $f_{\mathrm{odd}}$ and $f_{\mathrm{even}}$ aiming to relate the pixel position in the acquisition frame (*i*,*j*) with the resulting viewing angles $\theta_{H}$ and $\theta_{V}$. They are defined in Equation (9) and shown in Figure 3b.

Following the literature about distortion mapping [32,33,34], different nonlinear equations were proposed and tested along the development of this work and are presented in the following Section 3.2. Defining $\tilde{𝚤}=\left(i-N_{V}/2\right)$ and $\tilde{𝚥}=\left(j-N_{H}/2\right)$ and both $\vec{A}$ and $\vec{B}$ as the vectors containing the set of parameters for both odd and even horizontal scanning directions respectively, the two viewing angles may be expressed, without loss of generality, as:

(9) $$f:\left(i,j\right)\overset{\vec{X}}{\mapsto }\left(\theta_{H},\theta_{V}\right)\left\{\begin{array}{cc} \; & \left[\begin{array}{c} \theta_{H}\left(i,j\right)\\ \theta_{V}\left(i,j\right) \end{array}\right]{}^{odd}=f\left(\tilde{𝚤},\tilde{𝚥},\vec{A}\right)\equiv f_{\mathrm{odd}}\\ \; & \left[\begin{array}{c} \theta_{H}\left(i,j\right)\\ \theta_{V}\left(i,j\right) \end{array}\right]{}^{even}=f\left(\tilde{𝚤},\tilde{𝚥},\vec{B}\right)\equiv f_{\mathrm{even}}. \end{array}\right.$$

Now, finding both mapping functions $f_{\mathrm{odd}}$ and $f_{\mathrm{even}}$ reduces to solving a non-linear least squares system (NLSQ) for a set of *M* control viewing angles $\theta_{H}^{m}$ and $\theta_{V}^{m}$ measured at different pixel positions $\left(i^{m},j^{m}\right)$, so both vector parameters $\vec{A}$ and $\vec{B}$ minimize an objective function g. Our suggested objective function is based on the angular error between the control viewing angles and the estimated ones at the measured pixel positions on the image using the mapping functions $f_{\mathrm{odd}}$ and $f_{\mathrm{even}}$, as expressed below.

(10) $$\begin{array}{cc} \{\vec{A},\vec{B}|\min_{\vec{X}}||g_{k}\left(i^{m},j^{m},\vec{X}\right)||{}_{2}^{2} & =\min_{\vec{X}}\left|\right|{\left[\begin{array}{c} \theta_{H}^{m}\\ \theta_{V}^{m} \end{array}\right]}_{k}-f_{k}\left(i^{m},j^{m},\vec{X}\right)\left|\right|{}_{2}^{2}=\\ & =\min_{\vec{X}}\sum_{m=1}^{M}\left|\right|{\left[\begin{array}{c} \theta_{H}^{m}-\theta_{H}\\ \theta_{V}^{m}-\theta_{V} \end{array}\right]}_{k}\left|\right|{}_{2}^{2}\;\forall m=1,..,M\;\mathrm{with}\;k=\mathrm{odd},\mathrm{even}\}. \end{array}$$

### 3.2. Distortion Mapping Equations

Let us now introduce the three different sets of mapping functions $f_{k}$ (with k=odd,even) that have been proposed and tested during this work. As previously commented, the mapping functions aim to model the discussed angular variation that causes the FOV distortion. Thus, non-linear terms must be added to the ideal linear case presented in Equation (8) where the angular resolution is constant.

According to this, the linear term has been expanded up to a third-order polynomial in order to include the effect of the varying angular velocity and acceleration of the MEMS. This expansion is common to all the proposed mapping functions and relates exclusively the effect on the scanning direction, horizontal or vertical, caused by its corresponding pixel direction on the image.

On one hand, $\theta_{H,0}$ and $\theta_{V,0}$ apply for a global shift of the viewing angles in each direction. On the other hand, ${\tilde{\Delta \theta }}_{H}$ and ${\tilde{\Delta \theta }}_{V}$ can be considered as the mean angular resolution as if the FOV was homogeneous. Then, the following non-linear terms are related to the MEMS scanning dynamics, being $\omega_{H}$ and $\omega_{V}$ related to the MEMS angular velocities due to their even symmetry whereas $\Omega_{H}$ and $\Omega_{V}$ are related to its angular acceleration because of their odd symmetry.

Firstly, an equation similar to the widely used optical distortion model [35] is proposed. This model introduces a radial distortion with terms proportional to the radial distance to the center of the image plus a couple of cross-correlated terms with a distortion center in pixels known as the tangential distortion. However, since the MEMS dynamics is asymmetric, we have introduced introduced different cross-correlated terms following the literature about distortion mapping for optical displays [32,33,34] that provide a higher degree-of-freedom (DOF) to the cross-effect of the scanning directions than the traditional imaging distortion model used in the first approach.

Then, the second and the third proposed mapping functions contain the same number of cross-correlated terms, three in particular, but they differ in the DOF given to the optical distortion centers in pixels. Whereas the second one presents a unique distortion center for all the terms, the third one has different individual distortion centers for each term becoming the mapping function with the highest DOF possible.

Once the common part of the three proposed mapping functions has been discussed as well as the inclusion of the cross-correlated terms, let us now introduce them separately in detail. Recall that the pixel positions are normalized as $\tilde{𝚤}=\left(i-N_{V}/2\right)$ and $\tilde{𝚥}=\left(j-N_{H}/2\right)$.

*Map 1: Optical-Like Mapping*

Defining the radius as $\tilde{r}={\tilde{𝚤}}^{2}+{\tilde{𝚥}}^{2}$, the radial $R_{n}$ and tangential $P_{n}$ traditional optical coefficients are applied with a common distortion center on the image $i_{c}$ and $j_{c}$.

(11) $$f_{k}^{M1}:\left(i,j\right)\overset{\vec{X}}{\mapsto }\left(\theta_{H},\theta_{V}\right)\left\{\begin{array}{c} \begin{array}{cc} \theta_{H}\left(i,j\right)= & \theta_{H,0}+{\tilde{\Delta \theta }}_{H}\left(\tilde{𝚥}+j_{c}\right)+\omega_{H}{\left(\tilde{𝚥}+j_{c}\right)}^{2}+\Omega_{H}{\left(\tilde{𝚥}+j_{c}\right)}^{3}+\\ + & R_{1}\tilde{r}+R_{2}{\tilde{r}}^{2}+R_{3}{\tilde{r}}^{4}+P_{1}\left(\tilde{r}+2{\left(\tilde{𝚥}+j_{c}\right)}^{2}\right)+2P_{2}\left(\tilde{𝚥}+j_{c}\right)\left(\tilde{𝚤}+i_{c}\right) \end{array}\\ \begin{array}{cc} \theta_{V}\left(i,j\right)= & \theta_{V,0}+{\tilde{\Delta \theta }}_{V}\left(\tilde{𝚤}+i_{c}\right)+\omega_{V}{\left(\tilde{𝚤}+i_{c}\right)}^{2}+\Omega_{V}{\left(\tilde{𝚥}+i_{c}\right)}^{3}+\\ + & R_{1}\tilde{r}+R_{2}{\tilde{r}}^{2}+R_{3}{\tilde{r}}^{4}+2P_{1}\left(\tilde{𝚥}+j_{c}\right)\left(\tilde{𝚤}+i_{c}\right)+P_{2}\left(\tilde{r}+2{\left(\tilde{𝚤}+i_{c}\right)}^{2}\right). \end{array} \end{array}\right.$$

*Map 2: Cross Mapping*

Instead of using the traditional distortion coefficients, the cross-terms are introduced with more general functions but using the same common distortion center. $P_{H,n}$ apply for the cross-terms affecting $\theta_{H}$ whereas $P_{V,n}$ for $\theta_{V}$.

(12) $$f_{k}^{M2}:\left(i,j\right)\overset{\vec{X}}{\mapsto }\left(\theta_{H},\theta_{V}\right)\left\{\begin{array}{c} \begin{array}{cc} \theta_{H}\left(i,j\right)= & \theta_{H,0}+{\tilde{\Delta \theta }}_{H}\left(\tilde{𝚥}+j_{c}\right)+\omega_{H}{\left(\tilde{𝚥}+j_{c}\right)}^{2}+\Omega_{H}{\left(\tilde{𝚥}+j_{c}\right)}^{3}+\\ + & P_{H,1}\left(\tilde{𝚥}+j_{c}\right)\left(\tilde{𝚤}+i_{c}\right)+P_{H,2}{\left(\tilde{𝚥}+j_{c}\right)}^{2}\left(\tilde{𝚤}+i_{c}\right)+P_{H,3}\left(\tilde{𝚥}+j_{c}\right){\left(\tilde{𝚤}+i_{c}\right)}^{2} \end{array}\\ \begin{array}{cc} \theta_{V}\left(i,j\right)= & \theta_{V,0}+{\tilde{\Delta \theta }}_{V}\left(\tilde{𝚤}+i_{c}\right)+\omega_{V}{\left(\tilde{𝚤}+i_{c}\right)}^{2}+\Omega_{V}{\left(\tilde{𝚤}+i_{c}\right)}^{3}+\\ + & P_{V,1}\left(\tilde{𝚥}+j_{c}\right)\left(\tilde{𝚤}+i_{c}\right)+P_{V,2}{\left(\tilde{𝚥}+j_{c}\right)}^{2}\left(\tilde{𝚤}+i_{c}\right)+P_{V,3}\left(\tilde{𝚥}+j_{c}\right){\left(\tilde{𝚤}+i_{c}\right)}^{2}. \end{array} \end{array}\right.$$

*Map 3: Multi-Decentered Cross-Mapping*

Finally, rather than using a unique distortion center on the image for all functions, this equation provides freedom to every term to have a specific distortion center.

(13) $$f_{k}^{M3}:\left(i,j\right)\overset{\vec{X}}{\mapsto }\left(\theta_{H},\theta_{V}\right)\left\{\begin{array}{c} \begin{array}{cc} \theta_{H}\left(i,j\right)= & \theta_{H,0}+{\tilde{\Delta \theta }}_{H}\left(\tilde{𝚥}+j_{0}\right)+\omega_{H}{\left(\tilde{𝚥}+j_{\omega 0}\right)}^{2}+\Omega_{H}{\left(\tilde{𝚥}+j_{\Omega 0}\right)}^{3}+\\ + & P_{H,1}\left(\tilde{𝚥}+j_{P1}\right)\left(\tilde{𝚤}+i_{P1}\right)+P_{H,2}{\left(\tilde{𝚥}+j_{P2}\right)}^{2}\left(\tilde{𝚤}+i_{P2}\right)+P_{H,3}\left(\tilde{𝚥}+j_{P3}\right){\left(\tilde{𝚤}+i_{P3}\right)}^{2} \end{array}\\ \begin{array}{cc} \theta_{V}\left(i,j\right)= & \theta_{V,0}+{\tilde{\Delta \theta }}_{V}\left(\tilde{𝚤}+i_{0}\right)+\omega_{V}{\left(\tilde{𝚤}+i_{\omega 0}\right)}^{2}+\Omega_{V}{\left(\tilde{𝚤}+i_{\Omega 0}\right)}^{3}+\\ + & P_{V,1}\left(\tilde{𝚥}+j_{P1}\right)\left(\tilde{𝚤}+i_{P1}\right)+P_{V,2}{\left(\tilde{𝚥}+j_{P2}\right)}^{2}\left(\tilde{𝚤}+i_{P2}\right)+P_{V,3}\left(\tilde{𝚥}+j_{P3}\right){\left(\tilde{𝚤}+i_{P3}\right)}^{2}. \end{array} \end{array}\right.$$

### 3.3. Calibration Pattern and Algorithm

**Figure 4:**
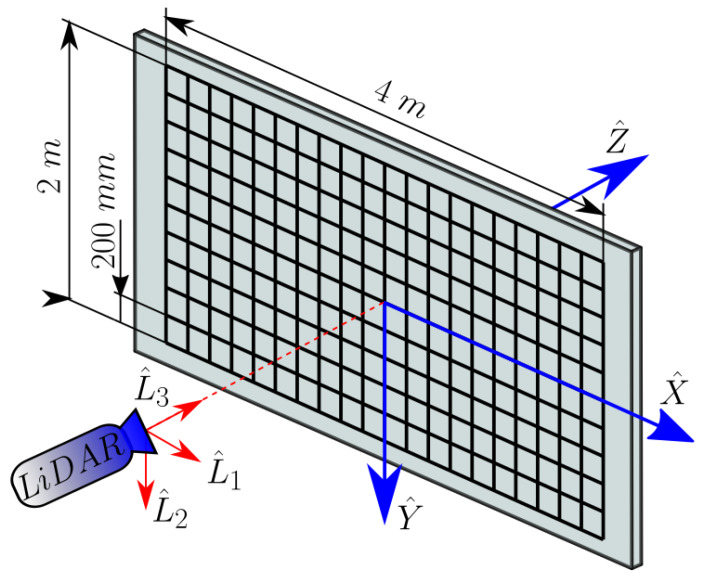
Scheme of the calibration pattern and position of the LiDAR.

In order to obtain the set of measurements, a regular grid of dimension 4 × 2 m with 200 mm width squares was constructed onto a planar wall with absorbent optical duct tape as depicted in Figure 4. Then, the LiDAR prototype is placed at a known *Z* distance facing the pattern so both viewing angles can be resolved using Equation (5) for all the scanned grid’s intersections.

**Figure 5:**
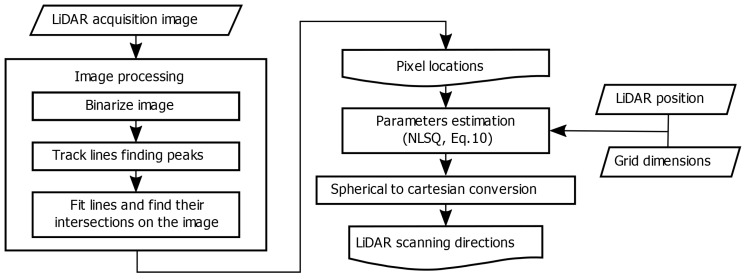
Step-by-step algorithm scheme for the estimation of the mapping function.

The scheme in Figure 5 describes the step-by-step procedure presented in pseudocode in Algorithm 1, which was implemented in practice using Matlab© (Algorithm 1 was implemented using standard Matlab© functions from the Signal Processing, Computer Vision and Optimization libraries.). Recall that, for the calibration method we propose, we consider a frame of the scanning optics as an image composed of even and odd scanning lines that we split in two images as discussed above and shown in Figure 3. Pixel locations from both odd and even images are obtained with sub-pixel resolution after doing line-detection using image processing, which includes a binarization of the image to work with the pixel locations corresponding to the black lines of the pattern, fitting them with two-dimensional lines on the image and calculating their intersections. Afterwards, these pixel locations in conjunction with the known grid dimensions and the LiDAR position, are introduced to an optimization solver according to the presented NLSQ problem of Equation (10) in order to estimate both $\vec{A}$ and $\vec{B}$ parameter vectors of the chosen mapping function. Finally, using the spherical description of the LiDAR scanning direction of Equation (7), the set of scanning directions are calculated from the mapped $\theta_{H}$ and $\theta_{V}$. Thus, the generated point cloud is calibrated over the whole system’s FOV. 

**Algorithm 1:** Image processing for obtaining the pixel locations of the lines’ intersections.

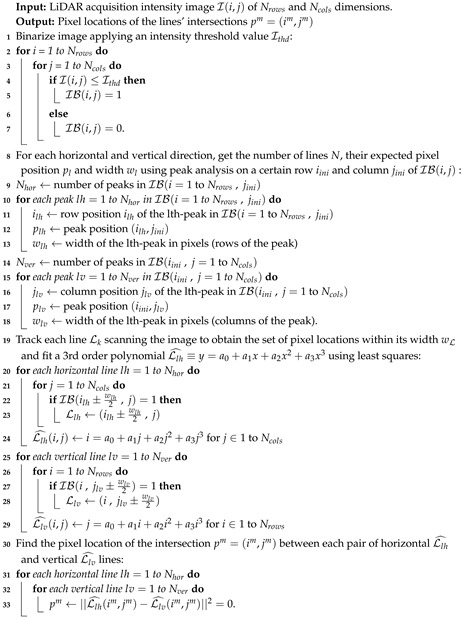



### 3.4. Prototypes

With the purpose of testing the method with different FOVs and distortions, two long range solid-state LiDAR prototypes from Beamagine S.L. with different FOV have been used. For the first one, we used a 30 × 20${}^{{}^{\circ}}$ FOV prototype with 300 × 150 pixels (45 k points) whereas for the second one, we used a 50 × 20${}^{{}^{\circ}}$ FOV with 500 × 150 pixels (75 k points).

**Figure 6:**
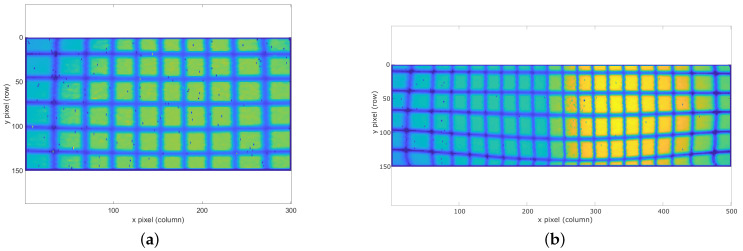
Acquired images of the calibration pattern with the (**a**) 30 × 20${}^{{}^{\circ}}$ FOV and (**b**) 50 × 20${}^{{}^{\circ}}$ FOV LiDAR prototypes.

Both scanning systems are based on MEMS technology, driving them with voltage control signals in both horizontal and vertical directions, the horizontal being the fastest one, resulting in a frame rate of 10 fps for both of them. As can be appreciated in Figure 6, which shows two images of the calibration pattern acquired with the two presented prototypes placed at the same distance, the second system presents a stronger distortion and asymmetry in MEMS dynamics due to its wider FOV.

## 4. Results

Once the model and the method for characterizing the scanning directions of a solid-state LiDAR system through mapping its angular resolution have been introduced and discussed, let us now present their results. Firstly, we are going to use the model for simulating the 30 × 20${}^{{}^{\circ}}$ FOV prototype and comparing it with experimental results. Later on, the calibration results for both prototypes are going to be presented and discussed.

### 4.1. Model Simulation

Firstly, the effect on the angular distortion of the combined effects of Snell’s law and the MEMS non-linear dynamics was studied. Using Equation (4) and the set of reference systems presented in Figure 1, the whole set of scanning directions ${\hat{s}}_{n}$ may be calculated from the value of the tilt angles of the MEMS mirror α and β. Afterwards, according to Equation (5) and Figure 2, both horizontal and vertical scanning angles are obtained applying a constant optical magnification over the whole FOV of the system for each direction.

**Figure 7:**
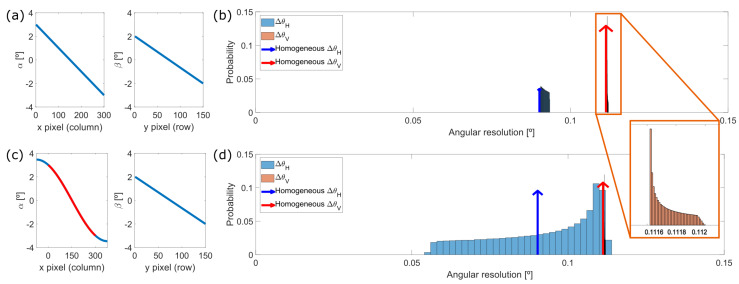
Simulated probability histogram of the angular resolution (**b**) and (**d**) for both scanning directions assuming, respectively, (**a**) linear motion of the MEMS and (**c**) harmonic motion of the MEMS in its fastest direction, being the red curve of α the cropped linear region of the whole motion shown in blue. Homogeneous resolution is calculated with $FOV_{H}=27.5^{{}^{\circ}}$ and $FOV_{V}=16.5^{{}^{\circ}}$.

In the case of the 30 × 20${}^{{}^{\circ}}$ FOV prototype, it creates a point cloud of 300 × 150 pixels and the MEMS is tilted ψ=−25${}^{{}^{\circ}}$ relative to the laser source according to specifications. The angular resolution in both directions of the scan is simulated using two methods: as a perfectly linear variation of the angles α and β (thus, including only the distortion effects due to Snell’s law), and, in a more realistic approach, modelling the fastest axis α of the mirror as a sinusoidal variation, as Figure 7a,c show respectively.

If the system was ideal and had a constant angular resolution, the probability of obtaining a given value of angular resolution would be a delta function at $FOV_{H}/N_{columns}$ and at $FOV_{V}/N_{rows}$, as depicted with an arrow in Figure 7b. The wider the histogram, the larger the deviation from the ideal homogeneous, linear case, as depicts the case Figure 7d.

Results show that even if the MEMS dynamics was completely linear, a slight broadening of the angular resolution would still appear due to the intrinsic non-linearity of Snell’s law. This widening of the delta function is significantly increased if the motion of the mirror is not linear, as shown in Figure 7d, where the non-linearity of the sinusoidal movement in α results in a widely distributed value of the $\Delta \theta_{H}$.

**Figure 8:**
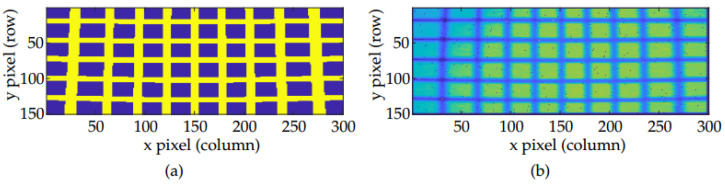
(**a**) Simulated and (**b**) experimental image of the grid pattern at 3.8 m using the 30 × 20${}^{{}^{\circ}}$ FOV prototype.

To further depict the effect, the resulting image from scanning the calibration pattern from a known distance of 3.8 m is simulated and qualitatively compared to an experimental capture. This comparison is going to become quantitative later on whilst comparing the angular resolutions across the whole FOV and in Section 4.3 as well. Both images are shown in Figure 8. Both of them present curves instead of straight lines as a result of fluctuation in angular resolution due to the different non-linearities in the system, as expected. Notice also how vertical lines are specially modified at the bottom of the image, where they bend towards the center, suggesting that not only the fast axis of the MEMS mirror, but also the slow one, present a non-linear behavior, as will be discussed later. Moreover, the asymmetric behaviour between odd and even rows of the image can be appreciated.

### 4.2. Calibration Results

**Figure 9:**
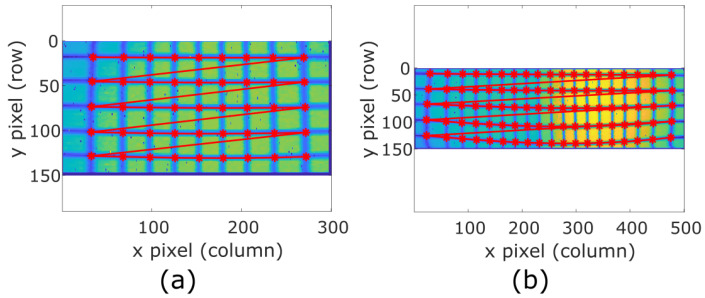
Grid control points on the odd image for both (**a**) 30 × 20${}^{{}^{\circ}}$ and (**b**) 50 × 20${}^{{}^{\circ}}$ FOV prototypes.

After simulating the model, we place the two prototypes being considered at a known distance of 3.8 m from the grid pattern described in Section 3, in order to test the calibration algorithm. The grid lines on the image are tracked and fitted, resulting in respectively 45 and 95 intersections for each LiDAR prototype as Figure 9 shows, which are the measured pixel positions $\left(i^{m},j^{m}\right)$.

**Figure 10:**
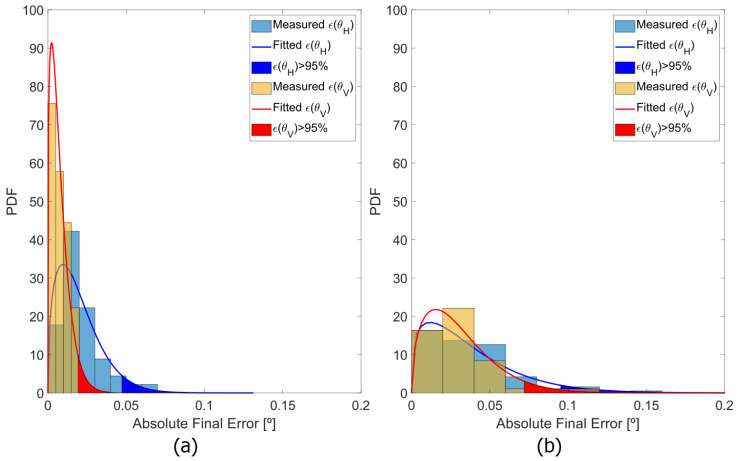
Probability Density Function (PDF) of both angular errors, (**blue**) horizontal and (**red**) vertical, of the odd lines for both (**a**) 30 × 20${}^{{}^{\circ}}$ FOV prototype and (**b**) 50 × 20${}^{{}^{\circ}}$ FOV prototype, using the mapping function Map 3 in Equation (13). The angular errors outside the 95% confidence interval are the filled areas.

According to Equation (10), the minimization algorithm is fed with the set of measured scanning angles $\left\{\theta_{H}^{m},\theta_{V}^{m}\right\}$ and their respective pixel positions $\left(i^{m},j^{m}\right)$ in the image in order to find the parameters of the three suggested mapping functions introduced in Section 3.2. In order to compare their performance, the error between the measured scanning angle and the estimated one using the mapping function is computed for all the control points. As figures of merit, we use the mean value of the angular error in all control points, its standard deviation and the value of angular error for which the Gamma function that fits its probability density function (PDF) is within a confidence value of 95%, as shown in Figure 10.

In addition, the rectangular region comprised inside the distorted FOV is considered as the homogeneous FOV of the system as discussed previously. Notice the fact that the rectangular region will be always tinnier than the designed FOV due to distortion, providing lower but indicative angular values. On Table 1, the obtained results for the figures of merit of each mapping function are presented, for both prototypes.

The table above shows that Map 1, the optical-like mapping function with radial and tangential coefficients, results in a less accurate estimation of the viewing angles than the other non-linear mapping functions tested for both LiDARs. The fact that optical distortion is assumed to have radial symmetry is considered to be the principal cause of this behavior. Nonetheless, despite their comparable performance with the 30 × 20${}^{{}^{\circ}}$ device, the Map 3 function shows improved results when the FOV of the system is wider and, as a consequence, distortion is stronger. It can be a result of having more degrees of freedom for the distortion optical centers of the nonlinear terms with respect to Map 2. However, notice that the homogeneous FOV is pretty similar for all mapping functions with respect to the prototypes’ designed FOV. Consequently, from now on the presented results correspond to the third mapping function Map 3.

Figure 10 shows the Probability Density Function (PDF) of the error committed in the mapping of the viewing angles for all the control points. On one hand, the PDF is narrower in the vertical direction for both LiDAR prototypes, as it is the slower direction of motion. On the other hand, the 30 × 20${}^{{}^{\circ}}$ FOV prototype presents smaller errors in both scanning directions using the same mapping function, which is coherent with the fact that the wider the FOV of the system, the higher the distortion it will present.

**Figure 11:**
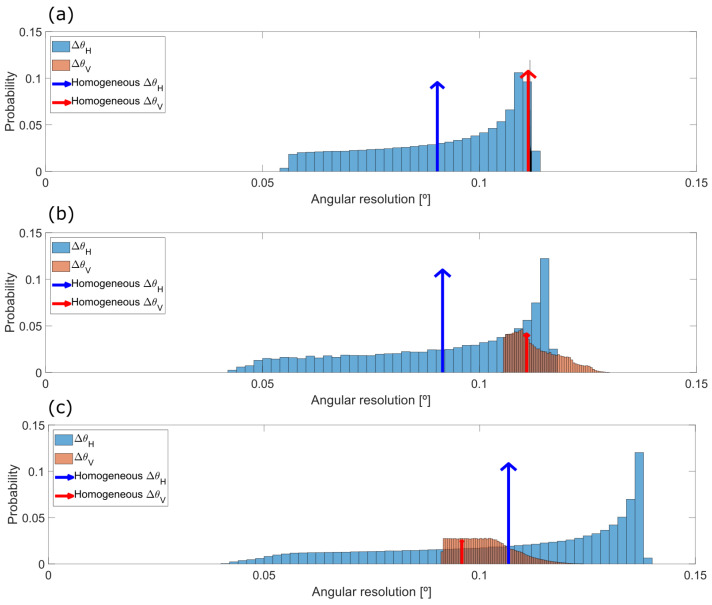
Comparison of both (**blue**) horizontal and (**red**) vertical resolution variation for the odd lines. (**a**) Simulation of the 30 × 20${}^{{}^{\circ}}$ FOV prototype with a sinusoidal dynamics for the fast scanning axis. (**b**) Results of the mapping function Map 3 for the 30 × 20${}^{{}^{\circ}}$ FOV prototype. (**c**) Results for the 50 × 20${}^{{}^{\circ}}$.

For the purpose of relating the analytic model with the obtained results from the proposed calibration method, Figure 11 compares the variation of the angular resolution for the 30 × 20${}^{{}^{\circ}}$ FOV prototype of the simulated sinusoidal MEMS dynamics for the fast axis, Figure 11a, against the variation obtained through the mapping function Map 3, Figure 11b. Additionally, it also presents the results of the same Map 3 for the 50 × 20${}^{{}^{\circ}}$ device in Figure 11c.

On the one hand, the above statement regarding the effect of the wider FOV on distortion can be observed again comparing Figure 11b against Figure 11c. On the other hand, it is easily observed that the mapped variations of angular resolution in the horizontal direction presented in Figure 11b are closer to the ones described in the simulated case Figure 11a than the vertical ones. This indicates that even the motion of the slow scanning direction is not completely linear.

**Figure 12:**
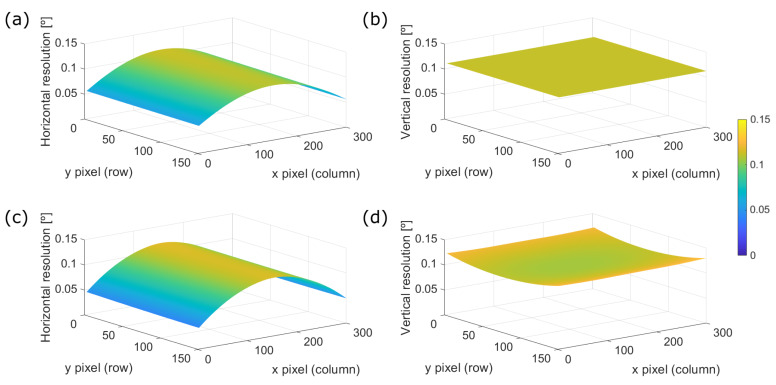
Comparison of respectively both horizontal and vertical angular resolution variations across the whole FOV of the 30 × 20${}^{{}^{\circ}}$ FOV prototype. (**a**,**b**) Sinusoidal dynamics for the fast scanning axis. (**c**,**d**) Results of the mapping function Map 3.

This is further shown in Figure 12, where the surface plots for the horizontal and vertical angular resolution variation maps of the 30 × 20${}^{{}^{\circ}}$ FOV prototype are respectively presented for its whole FOV. Figure 12a,b correspond to the simulated variations whereas Figure 12c,d to the measured ones using the mapping function Map 3. Ideally, we would expect a flat surface for the vertical angular resolution variation, which is the case Figure 12b shows, however, experimentally there exists a slight variation as Figure 12d presents.

**Figure 13:**
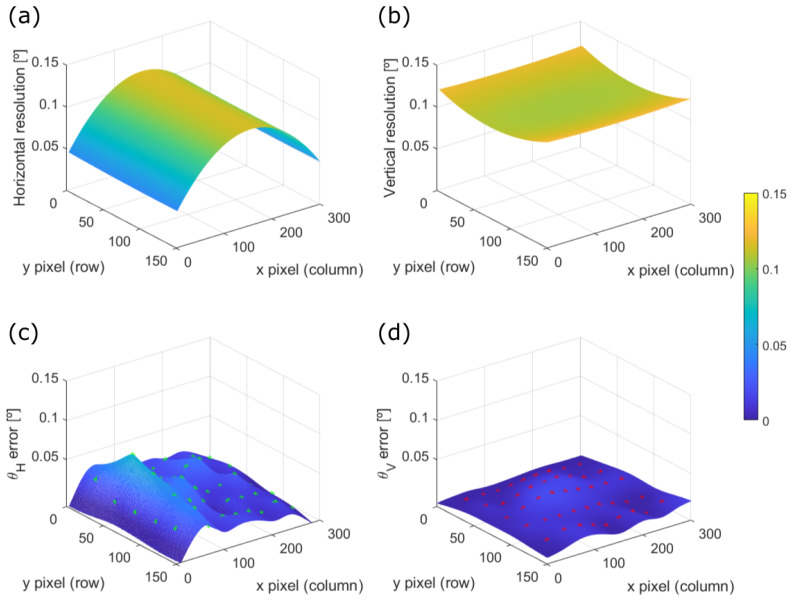
Results of the mapping function Map 3 for the 30 × 20${}^{{}^{\circ}}$ FOV prototype across its FOV. (**a**) Horizontal and (**b**) vertical angular resolution. (**c**) Horizontal and (**d**) vertical committed angular error.

The committed angular error on the control points previously presented in Figure 10 can be fitted with a biharmonic spline in order to interpolate such error across the whole FOV of the system as shows Figure 13c,d. Hence, we can compare the angular resolution variation on Figure 13a,b, for the horizontal and vertical directions respectively, with their corresponding committed errors of the mapping function Figure 13c,d.

It can be appreciated that the committed angular error is below the order of magnitude of the angular resolution of the LiDAR, even one order of magnitude below in some regions of the FOV. Thus, it can be concluded that the tested mapping functions are good approximations of the variable angular resolution across the FOV of a LiDAR device.

Finally, notice that both Figure 12 and Figure 13 show that the center of the point cloud has a lower density of points than the edges of the FOV because they are more separated, particularly in the horizontal direction. This result is compliant with the MEMS dynamics since in the middle of the horizontal scanning line its velocity is higher than in the edges, where the change in scanning direction takes place and damps the oscillation.

### 4.3. Impact on the Point Cloud

So far, results of the presented method have been expressed in terms of the scanning angles but, obviously such calibration errors have quantitative effects in the accuracy of the measured point cloud.

Using error propagation theory, the final point error $\vec{\delta }\left(i,j\right)={\left[\delta_{X},\delta_{Y},\delta_{Z}\right]}^{T}$ and its standard deviation $<\sigma_{k}>$, which is interpreted as its uncertainty, can be derived from the values of angular error $\epsilon_{H}$ and $\epsilon_{V}$ of the mapped scanning angles depicted in Figure 13. Hence, we can estimate which is the final lateral accuracy of the device in millimeters across its whole FOV, of course being aware that the distance error is proportional to the value of range measured. In addition, comparing them with the ones resulting from the ideal (but unreal) case of homogeneous angular resolution provides a quantitative measure of the improvement brought on by the method we propose.

**Figure 14:**
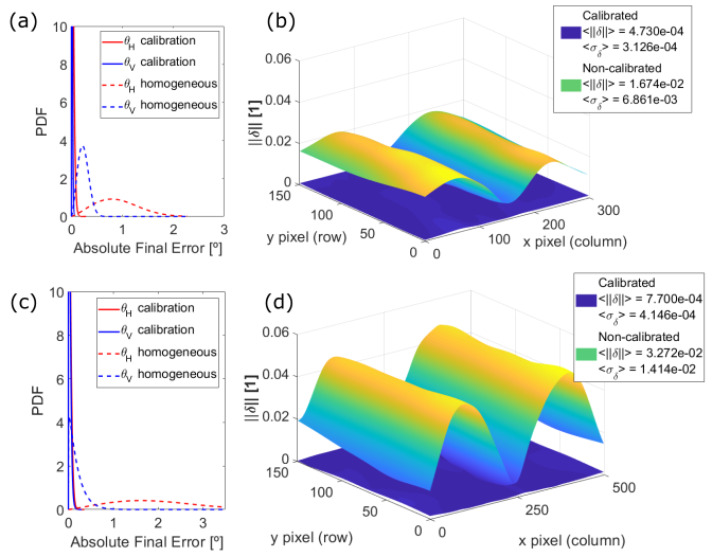
Improvement of the presented calibration for (**a**,**b**) the 30 × 20${}^{{}^{\circ}}$ and (**c**,**d**) the 50 × 20${}^{{}^{\circ}}$ FOV prototype, compared to their respective ideal cases of homogeneous angular resolution. (**a**,**c**) Probability density functions of the angular error, being (**red**) the horizontal and (**blue**) the vertical scanning direction of (**dashed line**) the non-calibrated and (**solid line**) the calibrated case. (**b**,**d**) Norm of the final point distance error.

With this aim, Figure 14 shows such comparisons of the initial non-calibrated and the final calibrated point cloud distortions for the two prototypes here described. Both PDFs in Figure 14a,c show the improvement in the angular error for the 30 × 20${}^{{}^{\circ}}$ FOV prototype and the 50 × 20${}^{{}^{\circ}}$ one respectively, whilst Figure 14b,d present the improvement in the distance error, which comes from propagating the angular error as previously commented. Notice that the final distance error $\vec{\delta }\left(i,j\right)$ is a 3-dimensional error but, for comparing a unique measurement, only the norm $\left|\right|\vec{\delta }\left(i,j\right)\left|\right|$ has been considered.

From Figure 14a,c, it can be observed that the dashed lines, which correspond to the ideal non-calibrated case, represent a wider angular error in both directions than the solid lines, which result from the calibration. Propagating such angular errors and comparing the norms of the distance error for each scanning position across the whole FOV of the prototypes, as Figure 14b,d depicts, show a very significant improvement of the calibration method against the non-calibrated cases. A larger FOV means also a larger error, calling for the relevance of a calibration process like the one presented here for LiDAR devices with larger FOVs.

As discussed earlier, the fastest scanning direction presents higher distortion and, consequently, larger error. Since this direction is the horizontal one and the non-lineal dynamics of the MEMS are greater at the extremes, the magnitude of the final distance error increases in the same direction as well. Figure 14 shows that, for both LiDAR prototypes, the PDf of the angular error is narrower after applying the suggested calibration. As a result, the final distance errors are one order of magnitude smaller compared to the ones obtained for the homogeneous angular case. In particular, the mean value of the error and its uncertainty across the whole FOV are reduced by a factor ∼1/40 and ∼1/30, respectively.

**Figure 15:**
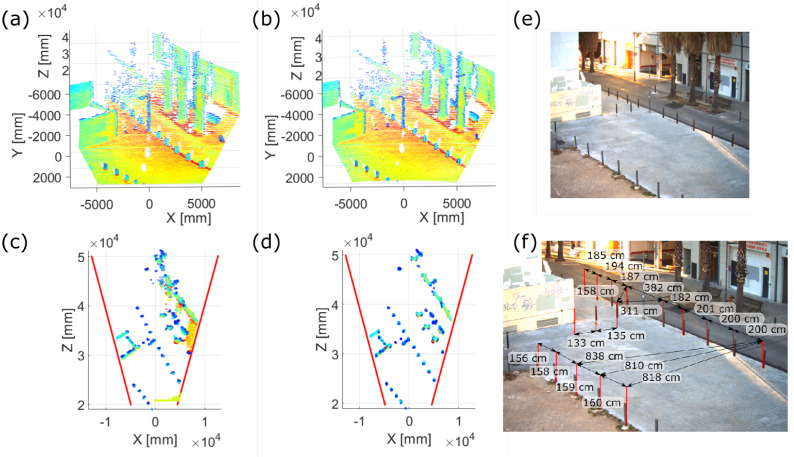
30 × 20${}^{{}^{\circ}}$ FOV prototype point clouds of (**e**) the scenario. (**a**) Previous calibration point cloud. (**b**) Presented calibration point cloud. (**c**) and (**d**) Orthogonal projection on the ground plane of (**a**) and (**b**) respectively. (**f**) Measured distances between road pins taken as the ground truth.

Empirically, these improvements can be directly observed in the measured point clouds. Figure 15 compares a point cloud obtained with the presented calibration method against one assuming constant angular resolution of the same scenario. The scene was captured using the 30 × 20${}^{{}^{\circ}}$ FOV prototype, which as commented is the one with smaller distance error. Even in that case, the road is not completely straight in the previous point cloud as Figure 15a shows.

This is better seen if the ground plane is rotated to the Y=0 plane so the orthogonal projections of the point cloud from above are depicted as both Figure 15c,d present. The projection of the rods forming the sidewalk barrier should be straight lines. On the one hand, the rods are visibly not forming a straight line in the non-calibrated point cloud, Figure 15c, what means that the lateral accuracy is poorer. On the other hand, they are also wider so the lateral precision is also inferior.

In order to provide quantitative indicators, the real distance between each road pin was physically measured with a measuring tape and taken as the ground truth shown in Figure 15f. Then, the computed centroid of each road pin on the point cloud is projected on the ground plane Y=0 and it is compared to the ground truth, obtaining a distance in metric units which represents the absolute metric error on the point cloud due to the FOV distortion.

The mean value of the considered absolute error results in values of several 100 mm for the non-calibrated case whereas, for the calibrated one, it is one order of magnitude smaller. In particular, for these captures that range between 20 and 40 m from the LiDAR, the mean lateral error and its uncertainty are of ±20 mm (±30 mm) for the calibrated case and of ±700 mm (±300 mm) for the other. These results closely match the expected lateral error and uncertainty shown in Figure 14, confirming the improvement of the calibration in one order of magnitude. It should be noted that at a range of 100 m, the corresponding lateral accuracies of the calibrated point cloud would be of ±48 mm (±32 mm) and ±77 mm (±42 mm) for the 30 × 20${}^{{}^{\circ}}$ and 50 × 20${}^{{}^{\circ}}$ FOV LiDAR prototypes respectively.

## 5. Conclusions

We have introduced a geometrical model for the scanning system of a LiDAR device based only in Snell’s law and its specific mechanics, what allows using the same procedure for other scanning techniques, either solid-state based or mechanical ones, as long as its scanning principle is carefully described. For instance, solid-state LiDAR systems based on Optical Phased Array devices (OPA) can relate their electrical control signals such as frequency with the final scanning direction using the presented approach.

In particular, for the solid-state MEMS mirror based system analyzed during this work, we have related its scanning direction to the tilt angles of the MEMS device, which has been tested to fit with the performance of a general solid-state LiDAR device including such elements. In addition, such a model may be used for characterizing the MEMS dynamics, which may be a first step towards either closed-loop control of the mirror oscillation, or, alternatively, towards the correction of the angular distortion of the optics and the MEMS dynamics by setting appropriate tilt angle dynamics for each scanning direction of the system.

Furthermore, the suggested geometrical model enables a calibration method which provides an accurate estimation of the varying angular resolution of the scanning system. We would like to emphasise that this method and the spherical description provided are generic to any scanning technique. Consequently, any other LiDAR device can use them and take advantage of their benefits, which are summarized below.

The presented work has been used to improve the accuracy of the measured point clouds, which will be more reliable as input for machine learning procedures. In particular, we have shown an improvement of one full order of magnitude in the accuracy of their lateral position measurements, preserving the shape of the objects on the final point cloud. Moreover, considering that the accuracy of the mapping functions is below the resolution of the system, other functions might be proposed but similar results will be obtained.

Overall, this work provides, for the first time in our knowledge, a general model for LiDAR scanning systems based on MEMS mirrors, which provides a simple calibration procedure based on the measurement of the angular errors of the scanner, which has been confirmed accurate, simple and useful for the characterization of such devices.

## Figures and Tables

**Table 1:** Mapping results of the tested prototypes. Notice that units are millidegrees (${}^{{}^{\circ}}$/1000) and the best performance is highlighted in **bold** for all figures except for the homogeneous FOV, which is in degrees and represents how far from the designed rectangular FOV is the distortion map.

Figure of Merit [Mdeg]	Dir.	30 × 20${}^{{}^{\circ}}$			50 × 20${}^{{}^{\circ}}$		
		Map 1	Map 2	Map 3	Map 1	Map 2	Map 3
Homogeneous FOV [${}^{{}^{\circ}}$]	Odd	27.55 × 16.32	27.47 × 16.54	27.47 × 16.52	52.89 × 13.96	53.19 × 14.44	53.34 × 14.38
	Even	27.25 × 16.44	27.46 × 16.64	27.45 × 16.63	53.08 × 13.33	53.07 × 14.27	53.07 × 14.21
Mean error	Odd	25 × 28	21 × 9	**20 × 8**	101 × 89	45 × 40	**37 × 31**
	Even	23 × 24	22 × 9	**22 × 9**	108 × 118	47 × 64	**46 × 37**
Standard deviation	Odd	24 × 23	14 × 5	**14 × 5**	66 × 97	34 × 32	**29 × 22**
	Even	24 × 18	14 × 7	**14 × 7**	73 × 110	37 × 48	**35 × 31**
Max. angular error (<95%)	Odd	79 × 70	**48 × 17**	47 × 19	239 × 274	117 × 103	**95 × 72**
	Even	77 × 60	48 × 25	**47 × 26**	265 × 324	117 × 165	**113 × 98**

## Appendix A

In this appendix the reader can find the detailed derivation of some of the key formulas used in previous sections.

### Appendix A.1. Vectorial Snell’s Law

Starting from Equation (2), $\hat{r}=\mu \hat{i}+\eta \hat{n}$, let us firstly apply the cross-product of the surface normal on both sides of the equation.

(A1) $$\hat{n}\times \hat{r}=\mu \left(\hat{n}\times \hat{i}\right)+\eta \left(\hat{n}\times \hat{n}\right)\Rightarrow |\hat{n}\left|\cdot \right|\hat{r}|\cdot \sin\left(\theta_{r}\right)\hat{A}=\mu \cdot |\hat{n}\left|\cdot \right|\hat{i}|\cdot \sin\left(\theta_{i}\right)\hat{B}$$

Since all ray vectors lay in the same plane, both $\hat{A}$ and $\hat{B}$ are parallel and using the scalar version of Snell’s law $\sin\theta_{r}/\sin\theta_{i}=n_{i}/n_{r}$, the expression for μ is found:

(A2) $$\mu =\frac{n_{i}}{n_{r}}$$

Secondly, let us apply the dot product of the reflected ray with itself in order to find the expression for η knowing that $\theta_{r}=-\theta_{i}$ so their cosines are equal:

(A3) $$\left(\mu \hat{i}+\eta \hat{n}\right)\cdot \left(\mu \hat{i}+\eta \hat{n}\right)=\mu^{2}+\eta^{2}+2\mu \eta \left(\hat{n}\cdot \hat{i}\right)=1\Rightarrow \eta^{2}+2\mu \left(\hat{n}\cdot \hat{i}\right)\eta +\left(\mu^{2}-1\right)=0$$

Solving this second order equation, it can be found the final expression for η:

(A4) $$\eta =\frac{-2\mu \left(\hat{n}\cdot \hat{i}\right)\pm \sqrt{4\mu^{2}{\left(\hat{n}\cdot \hat{i}\right)}^{2}-4\left(\mu^{2}-1\right)}}{2}=-\mu \left(\hat{n}\cdot \hat{i}\right)\pm \sqrt{1-\mu^{2}\left(1-{\left(\hat{n}\cdot \hat{i}\right)}^{2}\right)}$$

Thus, there are two solutions to the above equation: the positive one corresponds to the reflected case, while the negative one corresponds to the transmitted case. It can be easily deduced for a ray coming perpendicular onto the surface $\hat{n}=-\hat{i}$, so η=μ±1 and $\hat{r}=\mu \hat{i}+\left(\mu \pm 1\right)\hat{n}=\mp \hat{i}$. Finally, Equation (3) is the result of substituting these expressions for a reflected ray, thus the positive solution of Equation (A4), in Equation (2).

### Appendix A.2. Geometrical Model of MEMS Scanning

As described in Figure 1 in the paper, let us define three reference systems: one for the laser source $\left\{I\right\}=\left\{{\hat{i}}_{1},{\hat{i}}_{2},{\hat{i}}_{3}\right\}$, another one for the MEMS at its resting or central position $\left\{M\right\}=\left\{{\hat{m}}_{1},{\hat{m}}_{2},{\hat{m}}_{3}\right\}$, where ${\hat{m}}_{3}=\hat{n}$ is coincident with the surface normal at rest; and, finally, one more for the scanning $\left\{S\right\}=\left\{{\hat{s}}_{1},{\hat{s}}_{2},{\hat{s}}_{3}\right\}$ where ${\hat{s}}_{3}=\hat{r}$, the propagation vector of the beam when the MEMS is at its central position too. As it will be shown afterwards, this is the {M} reference system rotated again, matching ${\hat{s}}_{3}$ with the specular reflection of the incident ray. The aim is to express the MEMS normal and its incident ray in the same reference system in any scanning case so the set of scanning directions can be found by applying Equation (3).

Let us assume that {I} is in the origin of the Cartesian coordinate system, as depicted in Figure 1 (a) and that the incident laser beam is emitted in the *z* direction $\hat{i}=\hat{z}={\hat{i}}_{3}$. Additionally, the MEMS device is facing towards the laser source but tilted a certain angle ψ in the *x*-axis. Consequently, using the right-hand rule whilst setting ${\hat{m}}_{1}={\hat{i}}_{1}=\hat{x}$, this rotation can be expressed as:

(A5) $$R_{x}\left(\psi \right)=\left(\begin{array}{ccc} 1 & 0 & 0\\ 0 & \cos\left(\pi +\psi \right) & -\sin\left(\pi +\psi \right)\\ 0 & \sin\left(\pi +\psi \right) & \cos\left(\pi +\psi \right) \end{array}\right)=\left(\begin{array}{ccc} 1 & 0 & 0\\ 0 & -\cos\left(\psi \right) & \sin\left(\psi \right)\\ 0 & -\sin\left(\psi \right) & -\cos\left(\psi \right) \end{array}\right)$$

Consequently, the linear transformation from the MEMS reference system {M} to the source one {I}, without taking into account translations because the aim is to obtain its direction, is:

(A6) $${}^{\left\{M\}\to \{I\right\}}T=R_{x}\left(\psi \right)\cdot {}^{\left\{M\right\}}u$$

The driven tilts of the MEMS can be performed about both ${\hat{m}}_{1}$ and ${\hat{m}}_{2}$ MEMS axis. Let us define α as the angular tilt provoking an horizontal displacement in the FOV whereas β causes a vertical one. Hence, an α counterclockwise rotation can be expressed as a rotation about ${\hat{m}}_{2}$ whilst β about ${\hat{m}}_{1}$.

(A7) $$R_{m_{2}}\left(\alpha \right)\equiv R_{\alpha }=\left(\begin{array}{ccc} \cos\left(\alpha \right) & 0 & \sin\left(\alpha \right)\\ 0 & 1 & 0\\ -\sin\left(\alpha \right) & 0 & \cos\left(\alpha \right) \end{array}\right)\;\mathrm{and}\;R_{m_{1}}\left(\beta \right)\equiv R_{\beta }=\left(\begin{array}{ccc} 1 & 0 & 0\\ 0 & \cos\left(\beta \right) & -\sin\left(\beta \right)\\ 0 & \sin\left(\beta \right) & \cos\left(\beta \right) \end{array}\right)$$

With these rotation matrices, the MEMS surface normal in the laser reference system is defined as the result of applying β-α-ψ rotations, what defines the first rotation to be taken in the slow direction and matches a proper Euler angle rotation definition *x*-*y*-*x* in the fixed frame {M}:

(A8) $${}^{\left\{I\right\}}n=R_{\psi }\cdot R_{\alpha }\cdot R_{\beta }\cdot {}^{\left\{M\right\}}n\equiv {}^{\left\{M\}\to \{I\right\}}T\left(\alpha ,\beta ,\psi \right)\cdot {}^{\left\{M\right\}}n$$

As previously commented, the MEMS surface normal in its own reference system {M} coincides with the third vector of the basis ${\hat{m}}_{3}$ at the central position. Thus, the final expression for the MEMS normal in the reference system of the laser source {I} depending on the tilt angles results in:

(A9) $${}^{\left\{I\right\}}n\left(\alpha ,\beta ,\psi \right)={}^{\left\{M\}\to \{I\right\}}T\left(\alpha ,\beta ,\psi \right)\cdot \left[\begin{array}{c} 0\\ 0\\ 1 \end{array}\right]=\left[\begin{array}{c} \sin\left(\alpha \right)\cos\left(\beta \right)\\ \cos\left(\psi \right)\sin\left(\beta \right)+\sin\left(\psi \right)\cos\left(\alpha \right)\cos\left(\beta \right)\\ \sin\left(\psi \right)\sin\left(\beta \right)-\cos\left(\psi \right)\cos\left(\alpha \right)\cos\left(\beta \right) \end{array}\right]$$

Notice that if the small angle approximation is assumed for all angles (cos(x)∼1 and sin(x)∼x), rotations are commutative because any rotation order leads to ${}^{\left\{I\right\}}n={\left[\alpha ,\beta +\psi ,\beta \psi -1\right]}^{T}$.

Now that both vectors, the MEMS normal and the incident ray, are expressed in the same reference system, the expression for the scanning direction depending on the tilt angles can be found. Firstly, let us define γ(α,β,ψ) as the dot product between the MEMS normal and the incident ray.

(A10) $$\begin{array}{c} \gamma \left(\alpha ,\beta ,\psi \right)\equiv \left({}^{\left\{I\right\}}{\hat{n}}^{T}\cdot {}^{\left\{I\right\}}\hat{i}\right)={\left[\begin{array}{c} \sin\left(\alpha \right)\cos\left(\beta \right)\\ \cos\left(\psi \right)\sin\left(\beta \right)+\sin\left(\psi \right)\cos\left(\alpha \right)\cos\left(\beta \right)\\ \sin\left(\psi \right)\sin\left(\beta \right)-\cos\left(\psi \right)\cos\left(\alpha \right)\cos\left(\beta \right) \end{array}\right]}^{T}\cdot \left[\begin{array}{c} 0\\ 0\\ 1 \end{array}\right]=\\ =\sin\left(\psi \right)\sin\left(\beta \right)-\cos\left(\psi \right)\cos\left(\alpha \right)\cos\left(\beta \right) \end{array}$$

(A11) $${}^{\left\{I\right\}}\hat{s}=\;{}^{\left\{I\right\}}\hat{i}-\left(\gamma \left(\alpha ,\beta ,\psi \right)-\sqrt{\gamma {\left(\alpha ,\beta ,\psi \right)}^{2}}\right)\;{}^{\left\{I\right\}}\hat{n}$$

In particular, as previously commented, when the MEMS is at its rest position with α=β=0, the reflected ray results in:

(A12) $$\begin{array}{c} {}^{\left\{I\right\}}\hat{s}\left(0,0,\psi \right)=\left[\begin{array}{c} 0\\ 0\\ 1 \end{array}\right]-\left(-\cos\left(\psi \right)-\sqrt{{\left(-\cos\left(\psi \right)\right)}^{2}}\right)\left[\begin{array}{c} 0\\ \sin\left(\psi \right)\\ -\cos\left(\psi \right) \end{array}\right]=\left[\begin{array}{c} 0\\ 2\cos\left(\psi \right)\sin\left(\psi \right)\\ 1-2\cos{\left(\psi \right)}^{2} \end{array}\right]=\left[\begin{array}{c} 0\\ \sin\left(2\psi \right)\\ -\cos\left(2\psi \right) \end{array}\right] \end{array}$$

which is exactly the specular reflection of the incident ray with angle 2ψ. To sum up, let us write down the defined set of basis being aware that there is a translation with respect from the laser source and that they are defined for α=β=0.

(A13) $$\left\{I\right\}=\left\{\hat{i},\hat{j},\hat{k}\right\},\;\left\{M\right\}=\left\{\hat{i},-\left[\begin{array}{c} 0\\ \cos\left(\psi \right)\\ \sin\left(\psi \right) \end{array}\right],\left[\begin{array}{c} 0\\ \sin\left(\psi \right)\\ -\cos\left(\psi \right) \end{array}\right]\right\}\;\mathrm{and}\;\left\{S\right\}=\left\{\hat{i},-\left[\begin{array}{c} 0\\ \cos\left(2\psi \right)\\ \sin\left(2\psi \right) \end{array}\right],\left[\begin{array}{c} 0\\ \sin\left(2\psi \right)\\ -\cos\left(2\psi \right) \end{array}\right]\right\}$$

Thus, this is the set of reference basis that characterize the scanning of a LiDAR system.
